# Transcriptome analysis of genes and pathways associated with metabolism in *Scylla paramamosain* under different light intensities during indoor overwintering

**DOI:** 10.1186/s12864-020-07190-w

**Published:** 2020-11-10

**Authors:** Na Li, Junming Zhou, Huan Wang, Changkao Mu, Ce Shi, Lei Liu, Chunlin Wang

**Affiliations:** 1 School of Marine Science, Ningbo University, Ningbo, 315211 Zhejiang China; 2 Key Laboratory of Applied Marine Biotechnology, Ministry of Education, Ningbo University, Ningbo, 315211 Zhejiang China

**Keywords:** *Scylla paramamosain*, Light intensity, Metabolism, Transcriptome analysis

## Abstract

**Background:**

*Scylla paramamosain* is one of the commercially crucial marine crustaceans belonging to the genus *Scylla*, which is commonly distributed along the coasts of China, Vietnam, and Japan. Genomic and transcriptomic data are scarce for the mud crab. Light intensity is one of the ecological factors that affect *S. paramamosain* during indoor overwintering. To understand the energy metabolism mechanism adapted to light intensity, we analyzed the transcriptome of *S. paramamosain* hepatopancreas in response to different light intensities (0, 1.43, 40.31 μmol·m^− 2^·s^− 1^).

**Results:**

A total of 5052 differentially expressed genes were identified in low light group (LL group, 3104 genes were up-regulated and 1948 genes were down-regulated). A total of 7403 differentially expressed genes were identified in high light group (HL group, 5262 genes were up-regulated and 2141 genes were down-regulated). *S. paramamosain* adapts to different light intensity environments through the regulation of amino acids, fatty acids, carbon and energy metabolism. Different light intensities had a strong impact on the energy generation of *S. paramamosain* by influencing oxygen consumption rate, aerobic respiration, glycolysis/gluconeogenesis pathway, the citrate cycle (TCA cycle) and fatty acid degradation.

**Conclusion:**

Low light is more conducive to the survival of *S. paramamosain*, which needs to produce and consume relatively less energy to sustain physiological activities. In contrast, *S. paramamosain* produced more energy to adapt to the pressure of high light intensities. The findings of the study add to the knowledge of regulatory mechanisms related to *S. paramamosain* metabolism under different light intensities.

**Supplementary Information:**

**Supplementary information** accompanies this paper at 10.1186/s12864-020-07190-w.

## Background

There are many different stages in the life history of aquatic animals. Overwintering is an important life stage for aquatic animals living in some areas to adapt to cold winter conditions [1]. The process of overwintering determines the quality of parents and is a key factor in seedling development in the coming year [2]. There are many factors affecting the environment of aquatic animals during overwintering, such as salinity, temperature, food availability, and light.

In the aquatic environment, absorption and reflection of incident light is easily changed due to the presence of plankton, suspended particulate matter and soluble organic substances [3], which influences the light intensity of water. Therefore, light is one of the most variable water quality factors that directly or indirectly affects behavior [4, 5], survival and growth [6, 7], skin color [8, 9], digestion and immunity [10], fatty acid composition [11], and oxidative stress [12] in aquatic animals. Metabolism is an extremely important activity and has been studied extensively. Nalbach et al. demonstrated that the activity of the neuron-secreting cells in the eye stalk and the thoracic brain of crabs are closely related to light intensity [13]. Increased activity of neurosecretory cells under high light intensity promoted the activity of hormone-promoting hormones and metabolism-related hormones. Most shrimp and larvae react positively to light in the larval stage and a negative reaction to light in the adult stage [14]. Appropriate light conditions can improve the feeding rate of shrimp and crab larvae and promote growth and metamorphosis [15]. This phenomenon is particularly evident in the early developmental stages of shrimp and crab larvae.

*S. paramamosain* is an important marine crab species in China. This species has fast growth characteristics, large sized individuals, highly adaptable, delicious meat and high nutritional value [16]. *S. paramamosain* has played an important role in wild fisheries and aquaculture over the past few decades in China [17]. In 2018, the yield of *S. paramamosain* increased to 157,712 tons, accounting for 53.68% of the total marine crab aquaculture industry in China [18]. Although the culture of *S. paramamosain* has grown continuously for years, there are still very low yields, only 15–60 kg/667 m^2^ a year, which is unable to meet ever increasing market demand [19–21]. It is of urgent importance to improve the artificial breeding and breeding technology of *S. paramamosain* by improving the overwintering environment [22]. Therefore, understanding light intensity conditions required during the overwintering period are the focus of this study.

In recent years, with the development of molecular sequencing technology, non-parametric transcriptome sequencing technology has gradually become the main means of studying molecular mechanisms in biological research [23]. Transcriptome sequencing is the study of all mRNAs that a particular cell can transcribe under a certain functional state. For species without reference genomes, splicing small fragments out of Unigene (non repetitive sequence genes, a transcriptome database of the National Center for Biotechnology Information) and constructing reference sequences for subsequent analysis is an effective means of studying the molecular mechanisms and regulatory networks of non-parametric species. Analysis by Ding et al. identified key metabolic changes in the liver of the large yellow croaker (*Larimichthys crocea*) in response to acute hypoxia by transcriptome [24]. Wang et al. revealed the potential influencing mechanism of dietary astaxanthin on growth and metabolism in *Litopenaeus vannamei* by transcriptome [25]. Research carried out by Zou et al. described the change of aroma volatile metabolism in tomato (*Solanum lycopersicum*) fruit under different storage temperatures and 1-MCP treatment by transcriptome [26]. While there has been a lot of research on *S. paramamosain*, no studies have reported the applications of the transcriptome technique for investigating molecular mechanisms associated with metabolism under different light intensities. There has been no research to date on the molecular mechanism of light intensity on *S. paramamosain* metabolism.

In this study, the effects of different light intensities on *S. paramamosain* were investigated using transcriptome analysis, in particular the metabolic changes of gene expression under different light intensities. This study will assist in explaining the potential molecular mechanisms of *S. paramamosain* under different light intensities. These data may help to provide the necessary theoretical basis and technical support for breeding *S. paramamosain*.

## Results

### Differentially expressed genes (DEGs) in the hepatopancreas of *S. paramamosain*

A total of nine samples were used in this analysis. The Q30 of raw data for each sample was distributed from 93.00 to 94.09%, the effective data was distributed from 6.97 to 8.06 G, and the average GC content was 45.90% (Additional file 1: Table S3). Unigene 54,537 pieces were spliced with a total length of 60,890,086 bp and an average length of 1116 bp (Additional file 1: Table S2). As the genome sequencing of *S. paramamosain* has not yet been elucidated, after reads filtering, Trinity was used to perform de novo assembly with clean reads. Tgicl was used on cluster transcripts to remove abundance and obtain Unigenes.

**Fig. 1:**
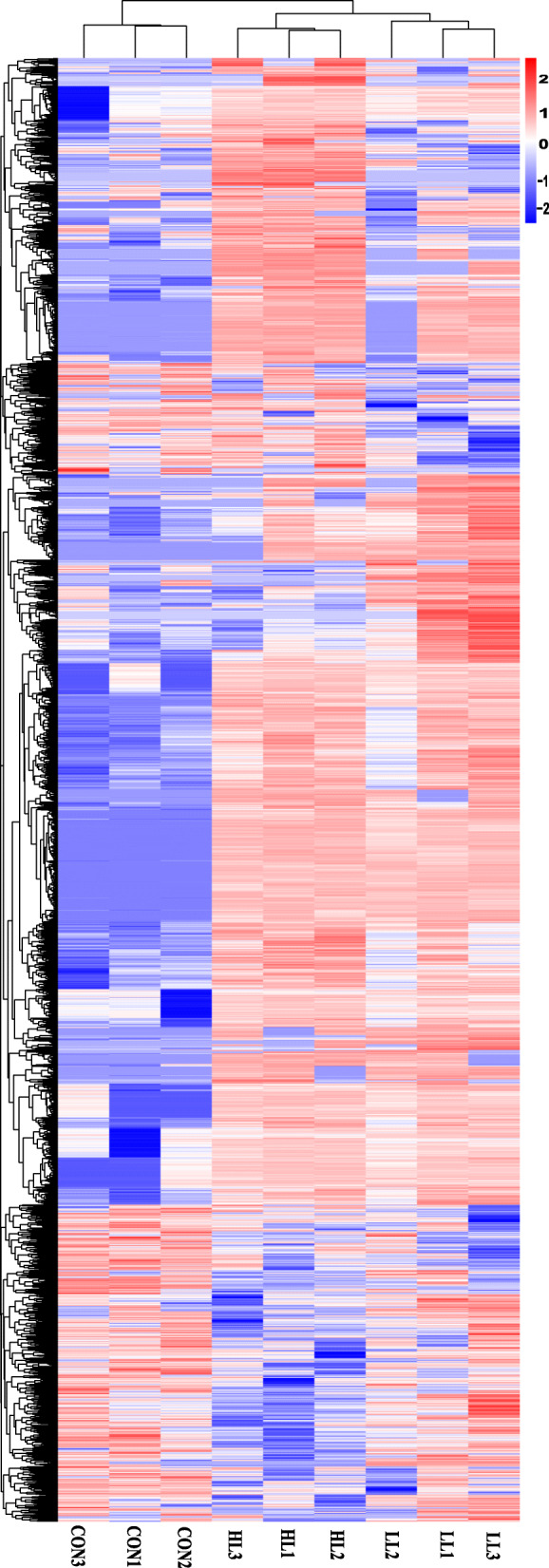
Hierarchical cluster analysis of DEGs in *S. paramamosain* hepatopancreas in the LL group vs the control (**a**) and the HL group vs the control (**b**). Different colors represent different relative abundance of genes, where red represents higher intensity and green represents lower intensity

The reads were compared to Unigene and the alignment was 90.63–93.10% (Additional file 1: Table S3). Stochasticity evaluation of sequencing data showed that the distribution of reads in the various parts of the gene was relatively uniform, indicating that the randomness of the break was good. The RPKM values of the individual genes determined under each 5% increment were compared to the expression levels of the final corresponding genes. The results differed by less than 15%, and therefore, sequencing data met quantitative requirements. The distribution of FPKM mean values for Unigenes ranged from 11.50 to 13.33 (Additional file 1: Fig. S1). Inter-sample correlation tests confirmed that the study was reliable and the sample selection was reasonable. Principal component analysis showed that each group of samples was distributed in different regions, and the same group of samples was concentrated spatially. Cluster analysis also showed that similar samples grouped together (Fig. 1).

Finally, high-quality transcripts were obtained and used as reference sequences. For database annotations of Unigenes, 16,049 (29.43%) genes were annotated into the NR library, 11,876 (21.78%) genes were annotated into the Swissprot library, 7881 (14.45%) genes were annotated into the KEGG library, 10,586 (19.41%) were annotated into the KOG library, 13,351 (24.48%) genes were annotated into the eggNOG library, 11,204 (20.54%) genes were annotated into the GO library, and 284 (0.52%) genes were annotated into the Pfam library (Additional file 1: Table S4). For functional annotation results, there were 54,287 SSRs predicted, including 25,440 Unigenes containing SSRs, 13,056 Unigenes containing more than one SSR and 11,842 composite SSRs (Additional file 1: Table S5). A total of 29,221 CDS sequences were predicted, of which 16,111 were predicted by the database comparison method and 13,110 were predicted by ESTScan. A total of 1672 Unigenes were annotated to the transcription factor database and distributed across 63 families.

**Fig. 2:**
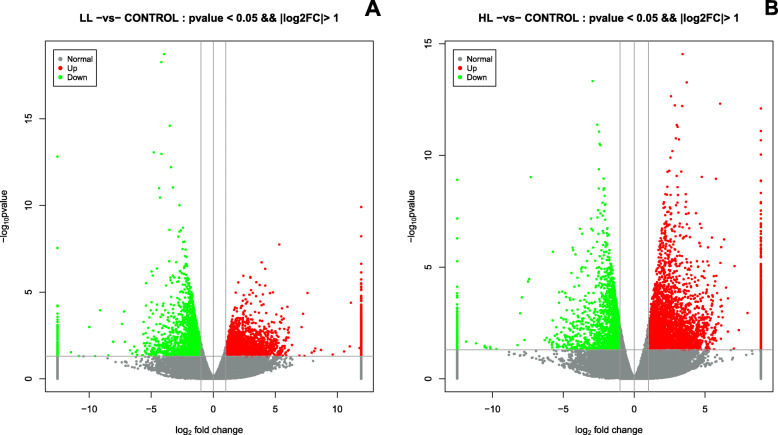
Volcano plots showing DEGs in *S. paramamosain* hepatopancreas in the LL group vs the control (**a**) and the HL group vs the control (**b**). Dots highlighted in red (FC > 1.2) and green (FC < 0.83) indicate genes significantly altered in abundance (*P* ≤ 0.05)

**Fig. 3:**
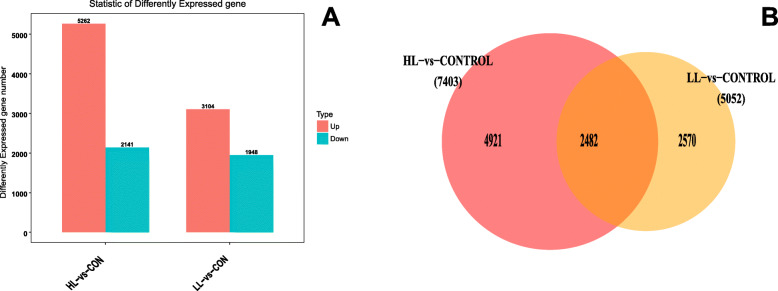
Distribution of differentially expressed genes (DEGs) with different light intensity effects in *S. paramamosain* hepatopancreas. **a** The number of DEGs (fold changes > 2 and Q-values < 0.05) under different light intensities. **b** Venn diagram of the up-regulated and down-regulated genes in the HL and LL groups

To compare the difference between the experimental group and control group in terms of the change in global proteomes in the hepatopancreas, volcano plots were created of *P*-value (−log10 *P*-value) versus the log2fold change for each gene analyzed. Accordingly, relative to the control group, the LL and HL group exhibited a change of a certain proportion of proteins (Fig. 2). A total of 5052 differentially expressed Unigenes were obtained between the LL group and the control, including 3104 up-regulated Unigenes and 1948 down-regulated Unigenes, while 7403 differentially expressed Unigenes were obtained between the HL group and the control, including 5262 up-regulated Unigenes and 2141 down-regulated Unigenes (*p* value< 0.05 & |log2FC| > 1) (Fig. 3a). Interestingly, 2482 differentially expressed Unigenes were intersected (Fig. 3b).

It is well known that carbon metabolism plays a key role in metabolic regulation. An important pathway in carbon metabolism is oxidative phosphorylation (ko00190), in which all significantly different genes are down-regulated. In the LL group, the three most highly down-regulated genes were NADH-ubiquinone oxidoreductase chain 3 (ID: P18930; 0.21-fold), NADH-ubiquinone oxidoreductase chain 2 (ID: P34848; 0.24-fold), and V-type proton ATPase subunit F (ID: Q1HQK8; 0.31-fold) (Table 1). In the HL group, the three most highly down-regulated genes were NADH-ubiquinone oxidoreductase chain 2 (ID: P34848; 0.10-fold), cytochrome c oxidase subunit 1 (ID: P00399; 0.17-fold), and NADH-ubiquinone oxidoreductase chain 4 (ID: Q34048; 0.17-fold) (Table 1). Two important pathways in energy metabolism are glycolysis / gluconeogenesis (ko00010) and the citrate cycle (TCA cycle, ko00020). In this process, only pyruvate dehydrogenase E1 component (ID: P49432) was significantly up-regulated. The fold change was 3.26 in the LL group and 2.64 in the HL group. In the LL group, the three most highly down-regulated genes were glyceraldehyde-3-phosphate dehydrogenase (ID: P56649; 0.25-fold), hexokinase type 2 (ID: Q9NFT7; 0.28-fold) and enolase (ID: P56252; 0.34-fold). In the HL group, the three most highly down-regulated genes were triosephosphate isomerase B (ID: Q90XG0 0.33-fold), aconitate hydratase (ID: Q99KI0; 0.37-fold), and glyceraldehyde-3-phosphate dehydrogenase (ID: P56649; 0.38-fold) (Table 1). Glycine, serine and threonine metabolism (ko00260) and beta-Alanine metabolism (ko00410) are important pathways in amino acid metabolism. 2-amino-3-ketobutyrate coenzyme A ligase (ID: O75600; LL: 2.13-fold, HL: 2.49-fold) and cystathionine gamma-lyase (ID: Q8VCN5; LL: 4.08-fold, HL: 16.10-fold) were up-regulated in both pathways. Betaine--homocysteine S-methyltransferase 1 (ID: Q5XGM3) was down-regulated in the LL group (0.53-fold) and up-regulated in the HL group (7.34-fold). In the LL group, the three most highly down-regulated genes were cytosolic non-specific dipeptidase (ID: Q96KP4; 0.13-fold), peroxisomal sarcosine oxidase (ID: Q29RU9; 0.16-fold), and aldehyde dehydrogenase (ID: P81178; 0.25-fold). In the HL group, the three most highly down-regulated genes were serine--pyruvate aminotransferase (ID: P41689; 0.19-fold), cytosolic non-specific dipeptidase (ID: Q96KP4; 0.21-fold) and D-3-phosphoglycerate dehydrogenase (ID: A5GFY8; 0.30-fold) (Table 1). Two important pathways in lipid metabolism are fatty acid elongation (ko00062) and fatty acid degradation (ko00071). Lysosomal thioesterase PPT2 (ID: O70489; LL: 3.47-fold, HL: 3.12-fold) and lysosomal thioesterase PPT2-B (ID: Q6GNY7; LL: 1.97-fold, HL: 3.00-fold) were up-regulated in both the LL and HL groups. In the LL group, fatty aldehyde dehydrogenase (ID: P47740) was the most down-regulated gene (0.31-fold), followed by alcohol dehydrogenase class-3 (ID: P79896, 0.38-fold) and hydroxyacyl-coenzyme A dehydrogenase (ID: Q16836; 0.44-fold) (Table 1). In the HL group, the three most down-regulated genes were hydroxyacyl-coenzyme A dehydrogenase (ID: Q16836; 0.35-fold), alcohol dehydrogenase class-3 (ID: P79896; 0.40-fold) and enoyl-CoA delta isomerase 2 (ID: Q9WUR2; 0.41-fold) (Table 1).

**Table 1:** The metabolic related differentially expressed genes (DEGs) identified in hepatopancreas of *Scylla paramamosain* under different light intensity by transcriptome

Swissprot annotation	Swissprot ID	LL/Control		HL/Control	
		FoldChange	*P* val	FoldChange	*P* val
**Oxidative phosphorylation (ko00190)**					
NADH dehydrogenase [ubiquinone] 1 alpha subcomplex subunit 11	Q9D8B4	0.4244	0.0069	0.3038	4.64E-05
NADH dehydrogenase [ubiquinone] 1 alpha subcomplex subunit 12	O97725	0.5027	0.0320	0.4272	0.0143
NADH dehydrogenase [ubiquinone] 1 alpha subcomplex subunit 7	Q9Z1P6	0.4448	0.0117	0.4501	0.0075
NADH dehydrogenase [ubiquinone] 1 subunit C2	Q02827	0.5304	0.0372	0.3998	0.0007
NADH dehydrogenase [ubiquinone] 1 beta subcomplex subunit 4	P0CB71	0.4975	0.0300	0.4757	0.0405
NADH dehydrogenase [ubiquinone] 1 beta subcomplex subunit 7	Q02368	0.4824	0.0272	0.4284	0.0164
NADH dehydrogenase [ubiquinone] 1 beta subcomplex subunit 3	Q02365	0.4691	0.0283	0.3656	0.0098
NADH dehydrogenase [ubiquinone] 1 beta subcomplex subunit 8	Q02372	0.4436	0.0078	0.3871	0.0028
NADH dehydrogenase [ubiquinone] 1 beta subcomplex subunit 2	Q0MQC7	0.4078	0.0034	0.3620	0.0001
NADH dehydrogenase [ubiquinone] iron-sulfur protein 4, mitochondrial	Q9CXZ1	0.4943	0.0210	0.3934	0.0004
NADH dehydrogenase [ubiquinone] iron-sulfur protein 7, mitochondrial	O75251	0.4492	0.0101	0.3609	0.0006
NADH-ubiquinone oxidoreductase chain 4 L	P18934	0.3750	0.0089	0.3079	0.0023
NADH-ubiquinone oxidoreductase chain 6	P18933	0.3685	0.0005	0.2222	3.38E-09
NADH-ubiquinone oxidoreductase chain 4	Q34048	0.3959	0.0099	0.1789	0.0487
NADH-ubiquinone oxidoreductase chain 2	P34848	0.2425	0.0004	0.1031	0.0055
NADH-ubiquinone oxidoreductase chain 3	P18930	0.2116	4.61E-05	0.2649	0.0001
ATP synthase subunit beta	Q5ZLC5	0.5238	0.0250	0.4125	0.0004
ATP synthase subunit g	Q5RFH0	0.3822	0.0011	0.3295	0.0001
ATP synthase subunit gamma	O01666	0.5652	0.0488	0.4692	0.0028
ATP synthase subunit d	Q24251	0.4474	0.0082	0.3143	0.0004
ATP synthase subunit b	Q94516	0.5377	0.0353	0.4163	0.0007
ATP synthase subunit delta	P35434	0.4663	0.0110	0.3982	0.0040
ATP synthase-coupling factor 6	Q24407	0.5513	0.0446	0.4851	0.0045
ATP synthase lipid-binding protein	Q9U505	0.5480	0.0355	0.1381	0.0260
Cytochrome c oxidase subunit 6B1	P14854	0.4849	0.0155	0.4162	0.0036
Cytochrome c oxidase subunit 1	P00399	0.4428	0.0028	0.1772	1.59E-05
Cytochrome c oxidase subunit 3	P00417	0.3186	0.0002	0.2095	0.0002
V-type proton ATPase subunit F	Q1HQK8	0.3166	9.16E-05	0.3091	0.0001
V-type proton ATPase subunit e 2	Q8NHE4	0.4510	0.0203	0.2667	0.0004
Inorganic pyrophosphatase	O77460	0.3413	0.0011	0.3730	0.0006
**Glycolysis / Gluconeogenesis (ko00010)**					
Aldose 1-epimerase	Q9GKX6	0.4300	0.0136	0.5034	0.0424
Phosphoglycerate kinase 1	Q5NVB5	0.4353	0.0035	0.4346	0.0010
Enolase	P56252	0.3451	0.0003	0.4208	0.0014
Glyceraldehyde-3-phosphate dehydrogenase	P56649	0.2565	0.0006	0.3811	0.0061
Glucose-6-phosphatase	O42153	0.4234	0.0031	0.4551	0.0040
Triosephosphate isomerase B	Q90XG0	0.3375	0.0002	0.3313	1.10E-05
Acetyl-coenzyme A synthetase	Q9NR19	–	–	0.5993	0.0439
L-lactate dehydrogenase	Q95028	–	–	0.5656	0.0288
Pyruvate dehydrogenase protein X component	Q8BKZ9	–	–	0.4991	0.0309
Hexokinase type 2	Q9NFT7	0.2888	0.0282	–	–
**Citrate cycle (TCA cycle, ko00020)**					
Pyruvate dehydrogenase E1 component subunit beta	P49432	3.2622	0.0022	2.6429	0.0005
Malate dehydrogenase	Q5NVR2	0.5229	0.0204	0.4403	0.0009
Succinate--CoA ligase [ADP/GDP-forming] subunit alpha, mitochondrial	P13086	0.5567	0.0452	0.4452	0.0016
Malate dehydrogenase	Q3T145	0.3996	0.0010	0.4326	0.0006
Cytoplasmic aconitate hydratase	Q0VCU1	0.4902	0.0109	0.5738	0.0262
Isocitrate dehydrogenase [NADP]	Q04467	0.4560	0.0079	0.5210	0.0216
Dihydrolipoyllysine-residue succinyltransferase component of 2-oxoglutarate dehydrogenase complex	Q9D2G2	–	–	0.4379	0.0020
Succinate dehydrogenase cytochrome b560 subunit	P70097	–	–	0.4841	0.0074
Aconitate hydratase	Q99KI0	–	–	0.3799	0.0001
Fumarate hydratase	Q60HF9	–	–	0.5111	0.0082
ATP-citrate synthase	Q91V92	0.4900	0.0239	–	–
Pyruvate carboxylase	Q29RK2	0.4938	0.0171	–	–
**Glycine, serine and threonine metabolism (ko00260)**					
Serine dehydratase-like	Q96GA7	0.4796	0.0457	0.3862	0.0043
Glycine cleavage system H protein	P23434	0.3558	0.0003	0.3919	0.0002
Phosphoglycerate mutase 2	Q32KV0	0.3546	0.0055	0.4024	0.0159
Serine--pyruvate aminotransferase	P41689	0.4186	0.0181	0.1968	2.98E-05
D-3-phosphoglycerate dehydrogenase	A5GFY8	0.3899	0.0042	0.3054	2.12E-05
2-amino-3-ketobutyrate coenzyme A ligase	O75600	2.1360	0.0470	2.4998	0.0070
Cystathionine gamma-lyase	Q8VCN5	4.0899	0.0094	16.1023	7.93E-05
Peroxisomal sarcosine oxidase	Q29RU9	0.1632	2.73E-09	0.4559	0.0016
Betaine--homocysteine S-methyltransferase 1	Q5XGM3	0.5321	0.0447	7.3483	0.0184
**beta-Alanine metabolism (ko00410)**					
Aldehyde dehydrogenase	P81178	0.2589	3.45E-06	0.4912	0.0037
Cytosolic non-specific dipeptidase	Q96KP4	0.1312	0.0006	0.2145	0.0087
Spermidine synthase	Q64674	0.5022	0.0310	0.5562	0.0409
Enoyl-CoA hydratase	P14604	–	–	0.4812	0.0056
Alpha-aminoadipic semialdehyde dehydrogenase	Q2KJC9	0.4374	0.0041	–	–
**Fatty acid elongation (ko00062)**					
Lysosomal thioesterase PPT2	O70489	3.4756	0.0236	3.1206	0.0462
Lysosomal thioesterase PPT2-B	Q6GNY7	1.9706	0.0252	3.0013	1.27E-05
3-ketoacyl-CoA thiolase	Q3T0R7	0.4633	0.0070	0.4285	0.0006
Hydroxyacyl-coenzyme A dehydrogenase	Q16836	0.4400	0.0349	0.3574	0.0132
**Fatty acid degradation (ko00071)**					
Short/branched chain specific acyl-CoA dehydrogenase, mitochondrial	P45954	–	–	0.5031	0.0261
Aldehyde dehydrogenase family 9 member A1-A	Q7ZVB2	–	–	0.4922	0.0255
Enoyl-CoA delta isomerase 2	Q9WUR2	–	–	0.4154	0.0024
Glutaryl-CoA dehydrogenase	Q2KHZ9	–	–	0.5238	0.0112
Long-chain specific acyl-CoA dehydrogenase	P51174	–	–	0.5771	0.0348
Trifunctional enzyme subunit beta	Q99JY0	–	–	0.4686	0.0035
Alcohol dehydrogenase class-3	P79896	0.3836	0.0010	0.4089	0.0005
Fatty aldehyde dehydrogenase	P47740	0.3161	0.0193	–	–

### Functional annotation

**Fig. 4:**
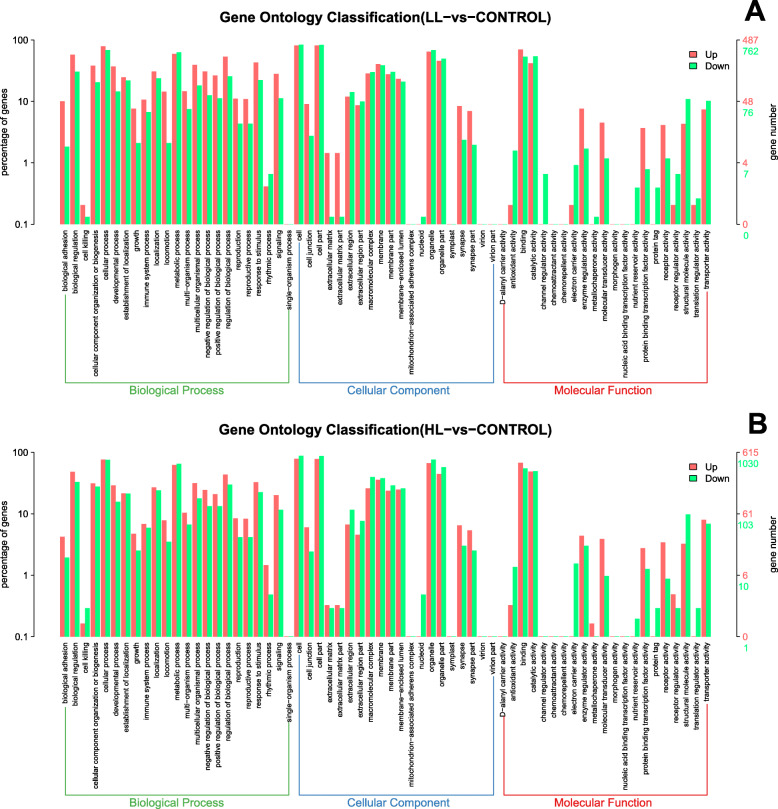
Gene Ontology (GO) classification showing DEGs in *S. paramamosain* hepatopancreas in the LL group vs the control (**a**) and the HL group vs the control (**b**)

Gene Ontology (GO) terms could be divided into three ontologies: cellular components, molecular function, and biological processes. In the LL group, 49 GO terms were assigned to the up-regulated group and 54 to the down-regulated group (Fig. 4a). In the HL group, 48 GO terms were assigned to the up-regulated group and 52 to the down-regulated group (Fig. 4b). A total of 23 processes were identified in the biological process category, comprising three biological processes (cellular process, metabolic process and biological regulation) as the most strongly affected in the hepatopancreas of *S. paramamosain* under different light intensities (Fig. 4). In the cellular component category, cells, cell parts, and membranes were most involved (Fig. 4). In the molecular function category, two items comprising binding and catalytic activities were most involved (Fig. 4).

### Metabolic related DEG pathway analysis

**Fig. 5:**
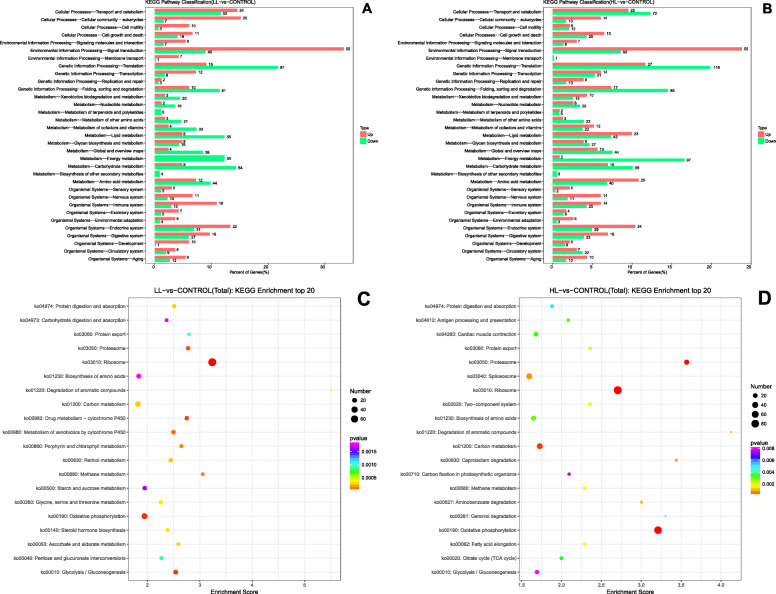
Kyoto Encyclopedia of Genes and Genomes (KEGG) pathway enrichment analyses of DEGs in *S. paramamosain* hepatopancreas hepatopancreas in the LL group vs the control (**a**&**c**) and the HL group vs the control (**b**&**d**)

KEGG pathway classification (Fig. 5) was performed according to the KEGG database website. All DEGs were graded into five categories according to their biological function, including cellular processes (LL, 150; HL, 175), environmental information processing (LL, 119; HL, 121), genetic information processing (LL, 198; HL, 309), metabolism (LL, 426; HL, 519) and organismal systems (LL, 227; HL, 247) (Fig. 5).

The pathways related to metabolism were subdivided into twelve subsets (level 2): xenobiotic biodegradation and metabolism (LL, 23; HL, 25), nucleotide metabolism (LL, 18; HL, 26), metabolism of terpenoids and polyketides (LL, 5; HL, 7), metabolism of other amino acids (LL, 24; HL, 26), metabolism of cofactors and vitamins (LL, 37; HL, 34), lipid metabolism (LL, 63; HL, 66), glycan biosynthesis and metabolism (LL, 27; HL, 36), global and overview maps (LL, 42; HL, 57), energy metabolism (LL, 55; HL, 99), carbohydrate metabolism (LL, 72; HL, 75), biosynthesis of secondary metabolism (LL, 4; HL, 3) and amino acid metabolism (LL, 56; HL, 65) (Fig. 5a&b and Additional file 1: Table S6).

Functional enrichment was also performed on DEGs according to the above KEGG pathway classification. The top 20 pathways showed that there were 15 and 12 pathways directly related to metabolism in the LL and HL groups, respectively. The most common metabolic pathways were biosynthesis of amino acids (ko01230), degradation of aromatic compounds (ko01220), carbon metabolism (ko01200), methane metabolism (ko00680), oxidative phosphorylation (ko00190) and glycolysis/Gluconeogenesis (ko00010) (Fig. 5c&d). The pathways related to metabolism in the LL group were pentose and glucuronate interconversions (ko00040), drug metabolism-cytochrome P450 (ko00982), starch and sucrose metabolism (ko00500), metabolism of xenobiotics by cytochrome P450 (ko00980), porphyrin and chlorophyll metabolism (ko00860), retinol metabolism (ko00830), glycine, serine and threonine metabolism (ko00260), steroid hormone biosynthesis (ko00140) and ascorbate and aldarate metabolism (ko00053) (Fig. 5c). The pathways related to metabolism in the HL group were caprolactam degradation (ko00930), carbon fixation in photosynthetic organisms (ko00710), aminobenzoate degradation (ko00627), geraniol degradation (ko00281), fatty acid elongation (ko00062) and the citrate cycle (TCA cycle) (ko00020) (Fig. 5d).

Seven important metabolic pathways were selected for further analysis: Glycine, serine and threonine metabolism (ko00260), beta-Alanine metabolism (ko00410), fatty acid elongation (ko00062), fatty acid degradation (ko00071), oxidative phosphorylation (ko00190), glycolysis / gluconeogenesis (ko00010) and the TCA cycle (ko00020) (Table 1). We focused on these pathways and the differential genes they contain.

### Validity of DEGs in transcriptomic data

**Fig. 6:**
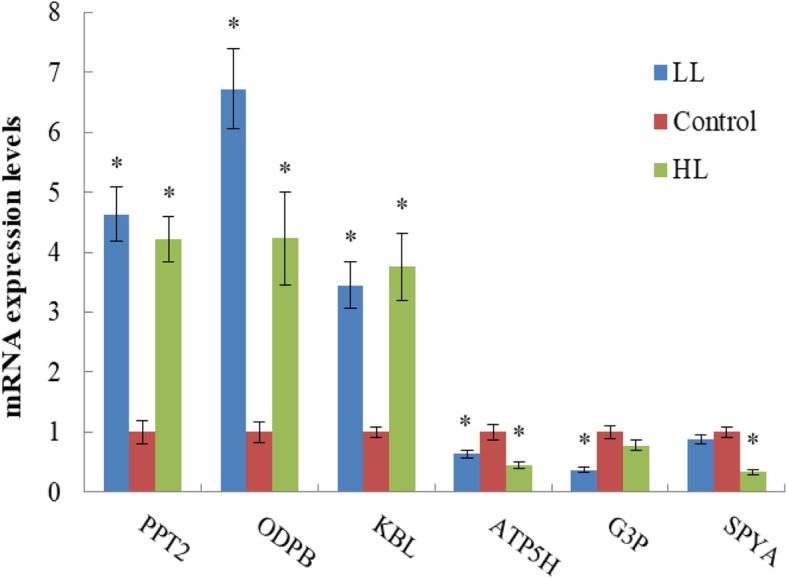
Quantitative real-time PCR (qRT-PCR) analysis of six differentially expressed genes in *S. paramamosain* hepatopancreas under different light intensities using the 2 ^−ΔΔCt^ method. Data are presented as means (± SD). Means denoted by * showed significant differences (*P* < 0.05). Definition of abbreviations: PPT2, lysosomal thioesterase PPT2; ODPB, pyruvate dehydrogenase E1 component subunit beta; KBL, 2-amino-3-ketobutyrate coenzyme A ligase; ATP5H, ATP synthase subunit d; G3P, Glyceraldehyde-3-phosphate dehydrogenase; SPYA, Serine-pyruvate aminotransferase

To validate the transcriptome results, qRT-PCR was used to check the transcript levels in six identified DEGs (PPT2, ODPB, KBL, ATP5H, G3P, and SPYA). As indicated by qRT-PCR, in the six genes selected in a random manner, the majority of the genes exhibited a similar transcription level expression to the transcriptome results in different light intensities, with the exception of G3P in the HL group and SPYA in the LL group (Fig. 6). The qRT-PCR results showed a high level of consistency with the transcriptome data obtained from transcriptome, indicating that the transcriptome results were reliable.

## Discussion

Light is an important and variable factor in both natural and farmed ponds [27]. *S. paramamosain* living in water is easily impacted by light intensity and the impacts of different light intensities on aquatic crustaceans have received some attention in recent years. One of the main functions of the hepatopancreas is metabolism [28, 29]. This study focuses on using transcriptome technology to investigate metabolism gene expression changes in the hepatopancreas of *S. paramamosain* in different light intensities. Four functional categories were selected for evaluating the dataset, mainly referring to carbohydrate metabolism, energy metabolism, amino acid metabolism, as well as lipid metabolism. The following sections will focus on discussing the biological relevance exhibited by the above-mentioned seven metabolic pathways.

### Effect of light intensity on energy metabolism of *S. paramamosain*

Among the energy metabolism pathways that were enriched, oxidative phosphorylation (ko00190) was the most affected. Five types of genes (NADH dehydrogenase, NADH-ubiquinone oxidoreductase, ATP synthase, cytochrome c oxidase, inorganic pyrophosphatase) were significantly down-regulated, and the fold change of the HL group was significantly higher than that of the LL group.

Mitochondria are the driving force for all living things to carry out life activities, and are the main sites for intracellular oxidative phosphorylation and formation of ATP which drives cellular functions [30]. Oxidative paosphorylation is a coupling reaction between the energy released by the body, ATP, and inorganic phosphate synthesis through the respiratory chain [31]. NADH-ubiquinone oxidoreductase and cytochrome c oxidase (COX) participate in the transfer of electrons to oxygen, and thus, are considered the key components of the respiratory chain [32]. Previous studies have shown that light affects oxygen consumption in the respiratory chain of crustaceans. Crayfish (*Procambarus clarkia* and *Procambarus digueti*) have a reduced oxygen consumption rate during long light periods [33]. The oxygen consumption rate of Chinese prawn and *Orconectes nais* was not affected by the light cycle [34]. The larvae of *Macrobrachium rosenbergii* have a high oxygen consumption rate in the dark, while lobster larvae and Chinese prawn exhibit high oxygen consumption under light conditions [35]. The oxygen consumption rate of *P. vannamei* was not affected by light intensity [36]. The average oxygen consumption rate of *Penaeus chinensis* in different shades of light was different under dark and light conditions. In this study, the change of NADH-ubiquinone oxidoreductase and COX genes showed that different light intensities affected the respiratory process of *S. paramamosain*. Significant down-regulation of NADH-ubiquinone oxidoreductase and COX genes indicated the reduced transfer of electrons to oxygen and a reduced oxygen consumption rate under light conditions. In addition, the oxygen consumption rate under high light intensity was lower than that under low light intensity.

The reaction catalyzed by the inorganic pyrophosphatase produces inorganic pyrophosphate, which relies on several important nucleotide triphosphates and is necessary for the synthesis of RNA, DNA, proteins, and lipids. While providing Pi for biomolecules, the inorganic pyrophosphatase can synthesize ATP, a terminal product of cellular energy metabolism [37]. NADH dehydrogenase which is produced by reduced coenzyme NADH is involved in many biochemical metabolic processes, such as glycolysis, the Krebs cycle, and beta oxidation [38]. This coenzyme contains electrons with a high electrode potential, which release a lot of energy when oxidized [39]. The results of the current study showed that inorganic pyrophosphatase genes in both experimental groups were significantly down-regulated, indicating that the amount of ATP synthesis was significantly reduced under light conditions. This conclusion can also be illustrated from the negative regulation of NADH dehydrogenase complex and ATP synthase subunit enrichment under light conditions in the current study.

### Effect of light intensity on carbohydrate metabolism of *S. paramamosain*

The hepatopancreas is the main organ of carbohydrate metabolism in crustaceans, and facilitates the maintenance of blood glucose levels through four metabolic compounds and the metabolic pathways they participate in; glycosylation, glycogenolysis, glycolysis and gluconeogenesis [40, 41]. According to enrichment analysis of the KEGG pathway, two important pathways related to carbohydrate metabolism were found: glycolysis/gluconeogenesis (ko00010) and the the citrate cycle (TCA cycle) (ko00020). Many of the genes in these pathways changed significantly, indicating that carbohydrate metabolism was of great significance for *S. paramamosain* to adapt to different light conditions.

Because the hepatopancreas stores a limited amount of glucose or glycogen, once this has been used up the body needs to synthesize glucose through gluconeogenesis to maintain normal energy requirements. During the process of gluconeogenesis, glucone-6-phosphatase catalyzes the conversion of non-sugar substances (such as lactic acid, pyruvate, propionic acid, glycerol, etc.) into glucose or glycogen [42, 43]. Triosephosphate isomerase, an important enzyme of the glycolysis/gluconeogenesis pathway, interconverts dihydroxyacetone phosphate and glyceraldehyde-3-phosphate dehydrogenase (G3P) to prevent the accumulation of cell-toxic dihydroxyacetone phosphate [44]. Under light conditions, the glycolysis /gluconeogenesis-related enzymes, such as triosephosphate isomerase, G3P, L-lactate dehydrogenase, acetyl-coenzyme A (acetyl-coA) and glucone-6-phosphatase were significantly down-regulated, indicating that the synthesis pathway of glycolysis /gluconeogenesis was inhibited to some extent. It can be seen from the oxidative phosphorylation pathway of energy metabolism that aerobic respiration is more vigorous under light conditions. This process produces carbon dioxide and water, releases energy and synthesizes large amounts of ATP by completely oxidizing and breaking down organic matter (usually decomposing glucose). This may be the main reason for inhibiting the glycolysis/gluconeogenesis pathway. In this study, pyruvate kinase gene expression was up-regulated by 9.7-fold in the HL group, which promoted the production of pyruvate which can be converted into acetyl-coA and enter the fatty acid biosynthesis pathway [45].

The citrate cycle (TCA cycle) is the third stage of catabolism, in which acetyl-coA is completely oxidized and decomposed into carbon dioxide [46]. The related genes, except for the pyruvate dehydrogenase (PDH) gene, were significantly down-regulated. PDH is one of the key enzymes in the central metabolic system. When pyruvate enters the TCA cycle, it first forms acetyl-coA under the action of PDH in the mitochondrial inner membrane, and then enters the TCA cycle [47]. The PDH gene was significantly up-regulated by 2.6-fold in the HL group and 3.2-fold in the LL group. However, there was no significant difference in acetyl-coA gene between the HL and LL groups, indicating that not enough pyruvate entered the TCA cycle to produce acetyl-coA. From the experimental results, the main genes that were down-regulated in this cycle were catalytic enzymes genes, such as malate dehydrogenase, isocitrate dehydrogenase, ATP-citrate synthase, aconitate hydratase, and fumarate hydratase. This indicates that light conditions limit the TCA cycle of the organism and the specific mechanism behind this requires further study.

### Effect of light intensity on amino acid metabolism in *S. paramamosain*

In terms of amino acid metabolism, two genes were significantly up-regulated in the two groups: 2-amino-3-ketobutyrate coenzyme A ligase and cystathionine gamma-lyase. Betaine--homocysteine S-methyltransferase 1 and sarcosine dehydrogenase were significantly up-regulated in the HL group, but significantly down-regulated in the LL group. From the results of this study, it can be seen that the regulation patterns of amino acids under different light intensities have similarities and differences. Cystathionine is a metabolic intermediate of sulfur-containing amino acids, which is involved in the conversion between cysteine and methionine. Cystathionine gamma-lyase acts on cystathionine so that cystathionine eliminates pyruvate and NH_3_ and produces homocysteine [48]. The fold change of GCL in the HL group (16.10-fold change) was significantly higher than that in the LL group (4.08-fold change), indicating that the HL group produced more homocysteine. Homocysteine has been widely studied in human diseases, but it has not been studied in crustaceans. Sarcosine usually exists in the form of creatine phosphate, and organisms mainly rely on ATP to provide energy under stress [49]. However, ATP reserves in the body are very small, so continuous synthesis is needed, and creatine phosphate can promote the synthesis of ATP. Creatine dehydrogenase is an enzyme that catalyzes chemical reactions, and its elevation significantly reduces the level of creatine. The sarcosine content in high light conditions was significantly higher than that in low-light conditions, which may be related to the fact that *S. paramamosain* requires more energy under high light conditions.

Some studies have shown that some glycosylated amino acids (such as alanine, glycine and serine) are decomposed in the hepatopancreas, and the carbon skeleton can be converted into metabolites such as pyruvate, ketoglutaric acid, succinyl coA, fumaric acid and oxaloacetic acid through catabolism under stress conditions, thus, affecting amino acid metabolism [24]. However, under opposite environmental conditions, these metabolites are restricted from entering the TCA cycle to produce ATP for energy or glucose through the gluconeogenic pathway. This conclusion was confirmed by a significant decrease of the gluconeogenic pathway. The combination of amino acid catabolism and gluconeogenic pathways may be a mechanism for adaptation to light intensity. However, the specific role of some amino acids needs further study to fully understand amino acid dynamics in different light intensities.

### Effect of light intensity on lipid metabolism in *S. paramamosain*

Lipids are the most important substrates for maintaining tissue function and are also important energy stores, providing energy for organisms [50]. In this study, KEGG analysis screened two key metabolic pathways related to the synthesis of fatty acids including fatty acid elongation and fatty acid degradation. A total of 14 DEGs were found to be involved in these two metabolic pathways, indicating that light intensity significantly affects lipids metabolism.

Elongation of fatty acids is an important biological process in the biosynthesis of polyunsaturated fatty acids [51, 52]. Polyunsaturated fatty acids play an important role in maintaining the relative fluidity of cell membranes and ensuring the normal physiological function of cells. In this study, four genes were found to be significantly correlated with the content of polyunsaturated fatty acids: lysosomal thioesterase PPT2, lysosomal thioesterase PPT2-B, 3-ketoacyl-CoA thiolase and hydroxyacyl-coA. The lysosomal thiesterase gene plays an important role in fatty acid elongation, but the specific mechanism has not been reported and needs further study. Thiesterase plays an important catalytic role in the synthesis and degradation of fatty acids. Thiesterases can be divided into two groups according to their different functions, in which 3-ketoacyl-CoA thiolase plays an important role in fatty acid decomposition [53]. The significant down-regulation of the 3-ketoacyl-CoA thiolase gene indicates that the solution rate of fatty acids decreases and more fatty acids might be accumulated under light conditions. In addition, different genes in fatty acid degradation pathways differed between high-light and low-light conditions, but were significantly down-regulated in both. This result indicates that fatty acid degradation was somewhat inhibited in the presence of light, but the specific mechanisms of these actions requires further study.

## Conclusions

In this study, we used RNA-sequencing to construct gene expression profiles related to metabolism mechanisms in *S. paramamosain.* To our knowledge, this is the first transcriptome study of these mechanisms in *S. paramamosain*. The results of this study revealed that glycine, serine and threonine metabolism, beta-Alanine metabolism, fatty acid elongation, fatty acid degradation, oxidative phosphorylation, glycolysis/gluconeogenesis, and the TCA cycle are involved in metabolism mechanisms in *S. paramamosain*. In conclusion, energy metabolism, amino acid metabolism, carbohydrate metabolism, and lipid metabolism are important means of regulation to cope with different light intensities. These results contribute to the existing knowledge of energy metabolism mechanisms in aquatic animals when adapting to different light intensities. These data may help to understand the molecular mechanisms of metabolism under different light intensities in *S. paramamosain* from a new perspective. We also suggested that low light intensity could be used for indoor overwintering.

## Methods

### Source, breeding and grouping of *Scylla paramamosain*

*S. paramamosain* individuals were collected from the sea near Xiangshan City (121 57 14 E, 29 28 29 N) and reared in crab apartment systems. The crabs were acclimatized in the carb apartments for 10 days prior to experimentation. And the crabs (290 ± 40 g) were randomly placed into nine groups, which were placed in nine crab apartments. Three groups of crab apartments comprised an experimental group: HL group (high light illumination with a photon flux density of about 40.31 μmol·m^− 2^·s^− 1^), LL group (low light illumination with a photon flux density of about 1.43 μmol·m^− 2^·s^− 1^), and the control group (full darkness). The light sources of each experimental group were white LED lights, simulating natural light with full spectrum. Peak light intensity was measured using a spectroradiometer. The light cycle was controlled by a timer (10 L: 14D), which turned the lights on at 07:00 every day and turned them off at 17:00. During the experiment period, the crabs were fed once daily (at 17:00) with *Ruditapes philippinarums*. The food debris and feces were cleaned at the outfall under the tanks at 07:00 the next day. The content of moisture, crude protein, crude fat and ash in whole organs (wet basis) of *Ruditapes philippinarums* were 77.21, 16.40, 1.46 and 3.01%, respectively [54]. Apart from light intensities, all other daily feeding and management operations were the same for all experimental groups during the trial.

The experiment was conducted in December 2018–March 2019 for a period of 4 months. After the experiment, the hepatopancreases of three crabs were collected and pooled to generate a single pooled sample. A total of 9 hepatopancreases (3 pooled samples) were obtained from each treatment group and used for further analyses. All samples were stored at − 80 °C.

### RNA extraction, library construction and sequencing

Total RNA was extracted with Trizol Reagent (Invitrogen, Shanghai, China) from 100 mg of hepatopancreas tissue following the manufacturer’s instructions. The sample was detected in 1.5% agarose gel electrophoresis. All RNA samples were of high quality (OD260/280 = 2.10–2.15, OD260/230 ≥ 2.0).

After digesting the DNA with DNase, eukaryotic mRNA was enriched using magnetic beads with Oligo (dT). A disruption reagent was added to break the mRNA into short fragments. Using the interrupted mRNA as a template, single-strand cDNA was synthesized using a six-base random primer, and then a two-stranded reaction system was used to synthesize double-stranded cDNA, which was purified using the kit. The purified double-stranded cDNA was subjected to end repair, a tail was added and the sequencing linker was ligated. The fragment size was then selected, and finally PCR amplification was performed. The constructed library was qualified with an Agilent 2100 Bioanalyzer and sequenced using an Illumina HiSeq X Ten sequencer to generate 150 bp double-ended data.

### Gene annotation and classification

After removing adaptor sequences, ambiguous N nucleotides (with a ratio of *N* > 10%) and low-quality sequences (with a quality score < 5), the remaining clean reads were assembled using Trinity software as described for de novo transcriptome assembly without a reference genome, and the longest copies of redundant transcripts were regarded as unigenes. Using diamond software, Unigenes were compared to the NR (NCBI non-redundant protein sequences, http://www.ncbi.nlm.nih.gov/), KOG (clusters of euKaryotic Orthologous Groups, http://www.ncbi.nlm.nih.gov/COG/), GO (the Gene Ontology, http://www.geneontology.org/), Swiss-Prot (http://www.ebi.ac.uk/uniprot/), and KEGG (Kyoto Encyclopedia of Genes and Genomes, http://www.genome.jp/kegg/) databases, and PFMER software was used to compare the Pfam database for unigene functional analysis.

### Differential gene identification, enrichment, and pathway analysis

Gene expression levels were positively correlated with their abundance. In transcriptome sequencing analysis, Unigene expression levels could be estimated by counting the sequencing reads of Unigenes. The reads count was directly proportional to the true expression level of the gene and also positively correlated with the length of the gene and the depth of sequencing. The uniplexed Unigene database was used to identify the abundance of each Unigene by sequence similarity alignment. Bowtie2 software was used to get the number of Unigene reads in each sample, and Express software was applied to calculate the Unigene expression FPKM value. The number of Unigene counts for each sample was normalized using DESeq software to calculate the difference multiple. The negative binomial distribution test was used to test the significance of the difference of read numbers. Finally, the differentially expressed Unigenes were screened based on the difference multiple and the difference significance test results. The threshold for screening differences was *p* value < 0.05 and a fold change > 2.

The differential expression of Unigenes was analyzed by GO enrichment and its function was described. The number of differential mRNAs included in each GO term was counted and the significance of the differential Unigene enrichment in each GO entry was calculated using the hypergeometric distribution test. The pathway analysis of the differential Unigenes was performed based on the KEGG database, and the significance of the differential Unigene enrichment in each pathway entry was calculated by the hypergeometric distribution test.

### Quantitative real-time PCR (qRT-PCR) analysis

Trizol reagent (Takara Bio, Otsu, Japan) was used to extract the total RNA from the hepatopancreas samples following the manufacturer’s instructions. Electrophoresis using 1.0% agarose gel helped to assess the quality of RNA. After the purification of total RNA, PrimeScript®RT Reagent Kit With gDNA Eraser (Takara Bio Inc., Shiga, Japan) was used to synthesize first-strand cDNA following the manufacturer’s instructions. ddH_2_O was used to dilute the cDNA obtained to 1:10, which was used as a template for RT-PCR. Table 1 lists the primer information. SYBR®Premix Ex Taq™ (QIGAN) was used for conducting the qRT-PCR amplifications with a 20-mL reaction mixture on the ABI7500 Real-Time PCR Detection System (Applied Biosystems). All of these experiments were conducted in triplicate. The comparative threshold cycle method (2^-△△CT^) was used to analyze the expression levels of target genes, taking β-actin as the reference gene. Each experiment involved three biological replicates. All the data underwent tests to meet the requirements for ANOVA, and a one-way ANOVA together with Tukey’s HSD test were carried out to analyze the data. All data were represented by the mean ± standard deviation. SPSS statistical software 22.0 was used for statistical analyses. *P* values less than 0.05 were considered to be statistically significant.

## Supplementary Information

**Additional file 1: Table S1.** The gene-specific primers used in this study, **Table S2.** Result statistics of splicing, **Table S3.** Summary for transcriptome sequencing data generated from nine cDNA libraries, **Table S4.** Summary of annotated genes in different databases, **Table S5.** Statistical table of SSRs, **Table S6.** Sub categorization of the metabolism group, **Figure S1.** Distribution of FPKM value in each sample.

## Data Availability

Raw Illumina sequences were deposited in the National Center for Biotechnology Information (NCBI) and our SRA records will be accessible with the following link after the indicated release date: https://www.ncbi.nlm.nih.gov/sra/PRJNA640880, SRA accession: PRJNA640880; Temporary Submission ID: SUB7645760.

## Abbreviations

GO: Gene Ontology
KEGG: Kyoto Encyclopedia of Genes and Genomes
LL: Low light intensity
HL: High light intensity
*S. paramamosain*: *Scylla paramamosain*
qRT-PCR: Quantitative real-time PCR
PPT2: Lysosomal thioesterase PPT2
ODPB: Pyruvate dehydrogenase E1 component subunit beta
KBL: 2-amino-3-ketobutyrate coenzyme A ligase
ATP5H: ATP synthase subunit d
G3P: Glyceraldehyde-3-phosphate dehydrogenase
SPYA: Serine--pyruvate aminotransferase
