# Supplementary material for: Transcriptome analysis of genes and pathways associated with metabolism in Scylla paramamosain under different light intensities during indoor overwintering
Source: BMC Genomics. 2020 Nov 10;21:775. doi: 10.1186/s12864-020-07190-w (PMC7654585; doi:10.1186/s12864-020-07190-w)
Supplement: Supplementary file 1 — Additional file 1: Table S1. The gene-specific primers used in this study, Table S2. Result statistics of splicing, Table S3. Summary for transcriptome sequencing data generated from nine cDNA libraries, Table S4. Summary of annotated genes in different databases, Table S5. Statistical table of SSRs, Table S6. Sub categorization of the metabolism group, Figure S1. Distribution of FPKM value in each sample. [file 12864_2020_7190_MOESM1_ESM.docx]

**Additional files**

**Table S1 Primers used for qRT-PCR.**

| Prmer name | Forward Primer(5'-3') | Reverse Primer(5'-3') | Purpose |
| --- | --- | --- | --- |
| PPT2 | TATCCATGGCGTCTGGGACT | CATACTGGCCAGCTTGAGGG | qRT-PCR |
| ODPB | TGTGTTCCGAGGACCCAATG | GCTGGGCTCTCAACAATCCT | qRT-PCR |
| KBL | TTGTGGCACTCAGACCATCC | GGTAAGGTCTTGCTCGCTGT | qRT-PCR |
| ATP5H | ATGGCAGCCCGAAGAGTC | CTCTGCACCCTCCTTGGC | qRT-PCR |
| G3P | ATGGCGTGTACAAGGGTGAG | TCAACCACGGACACATCAGG | qRT-PCR |
| SPYA | ATGTGTAACCTGCTGGAGCC | TGGTGGTATTTGCGAGGGAC | qRT-PCR |
| β-actin | GCCCTTCCTCACGCTATCCT | GCGGCAGTGGTCATCTCCT | qRT-PCR |

**Genes are abbreviated as follows**: Lysosomal thioesterase PPT2 (PPT2); Pyruvate dehydrogenase E1 component subunit beta (ODPB); 2-amino-3-ketobutyrate coenzyme A ligase (KBL); ATP synthase subunit d (ATP5H); Glyceraldehyde-3-phosphate dehydrogenase (G3P); Serine--pyruvate aminotransferase (SPYA).

**Table S2 Result statistics of splicing.**

| Term | All (>300bp) | >=500bp | >=100bp | N50 | Total_length | Max_Length | Min_Length | Average_Length |
| --- | --- | --- | --- | --- | --- | --- | --- | --- |
| Unigene | 54537 | 32514 | 17277 | 1876 | 60890086 | 21255 | 301 | 1116.49 |

**All (>300bp)**: Number of Unigene with length >= 300bp; **>=500bp**: Number of Unigene with length >=500bp; **>=1000bp**: Number of Unigene with length >=1000bp; **N50**: The N50 length is used to determine the assembly continuity, the higher the better. N50 is a weighted median statistic that 50% of the total length is contained in transcripts that are equal to or larger than this value; **Total_Length**: The read length of Unigenes; **Max_Length**: the longest length of Unigene; **Min_Length**: The shortest length of Unigene.

**Table S3 Summary for transcriptome sequencing data generated from nine cDNA libraries.**

| Sample | Raw_reads | Raw_bases | Clean_reads | Clean_bases | Vaid_bases(%) | Q30(%) | GC(%) |
| --- | --- | --- | --- | --- | --- | --- | --- |
| CONTROL1 | 55343246 | 8301486900 | 53193490 | 7748273699 | 93.34 | 93.69 | 45.69 |
| CONTROL2 | 50681538 | 7602230700 | 48770678 | 7096588572 | 93.35 | 93.70 | 46.73 |
| CONTROL3 | 49622514 | 7443377100 | 47955458 | 6972528373 | 93.67 | 94.09 | 45.76 |
| HL1 | 57682096 | 8652314400 | 55510188 | 8063552837 | 93.20 | 93.34 | 46.93 |
| HL2 | 54778946 | 8216841900 | 52572856 | 7611407371 | 92.63 | 93.00 | 45.70 |
| HL3 | 56136812 | 8420521800 | 54397854 | 7866049955 | 93.42 | 94.06 | 44.54 |
| LL1 | 53167384 | 7975107600 | 51237134 | 7429856855 | 93.16 | 93.53 | 45.57 |
| LL2 | 50602370 | 7590355500 | 48908268 | 7113470245 | 93.72 | 93.98 | 45.83 |
| LL3 | 56386788 | 8458018200 | 54198790 | 7863446770 | 92.97 | 93.22 | 46.33 |

**Sample**: Sample name; **RAW_reads**: The original reads number; **Raw_bases**: The raw sequencing quantity, or the number of bases; **Clean_reads**: The number of clean reads obtained after filtering; **Clean_bases**: The filtered sequencing quantity, i.e., the number of bases; **Valid_bases**: valid base percentage; **Q30**: The percentage of bases whose Phred value is greater than 30 in raw Bases; **GC**: The percentage of the total number of G and C in clean bases.

**Table S4 Summary of annotated genes in different databases.**

| Annotated Databases | Number of unigenes | Percentage(%) |
| --- | --- | --- |
| NR | 16049 | 29.43 |
| Swissprot | 11876 | 21.78 |
| KEGG | 7881 | 14.45 |
| KOG | 10586 | 19.41 |
| eggNOG | 13351 | 24.48 |
| GO | 11204 | 20.54 |
| Pfam | 284 | 0.52 |
| Total unigenes | 54537 | 100 |

**Table S5 Statistical table of SSRs**

| Unigene Number | Unigene Size | SSRs Number | Unigene Number(SSRs>=1) | Unigene Number(SSR>=2) | Compound SSRs Number |
| --- | --- | --- | --- | --- | --- |
| 54537 | 60890086 | 54287 | 25540 | 13056 | 11842 |

**Unigene Size**: Total base number of Unigene; **SSRs Number**: Number of predicted SSRs; **Unigene Number(SSR>=1)**: Number of Unigene containing SSRs; **Unigene Number(SSR>=2)**: Number of Unigene containing >=2 SSRs;

**Table S6 Sub categorization of the metabolism group.**

| Major Category | Subcategory | Gene number | Percentage(%) |
| --- | --- | --- | --- |
| Metabolism | Amino acid metabolism | 295 | 6.57 |
|  | Biosynthesis of other secondary metabolites | 31 | 0.69 |
|  | Carbohydrate metabolism | 389 | 8.66 |
|  | Energy metabolism | 213 | 4.74 |
|  | Glycan biosynthesis and metabolism | 235 | 5.23 |
|  | Lipid metabolism | 347 | 7.73 |
|  | Metabolism of cofactors and vitamins | 183 | 4.07 |
|  | Metabolism of other amino acids | 121 | 2.69 |
|  | Metabolism of terpenoids and polyketides | 54 | 1.2 |
|  | Nucleotide metabolism | 236 | 5.25 |
|  | Xenobiotics biodegradation and metabolism | 118 | 2.63 |


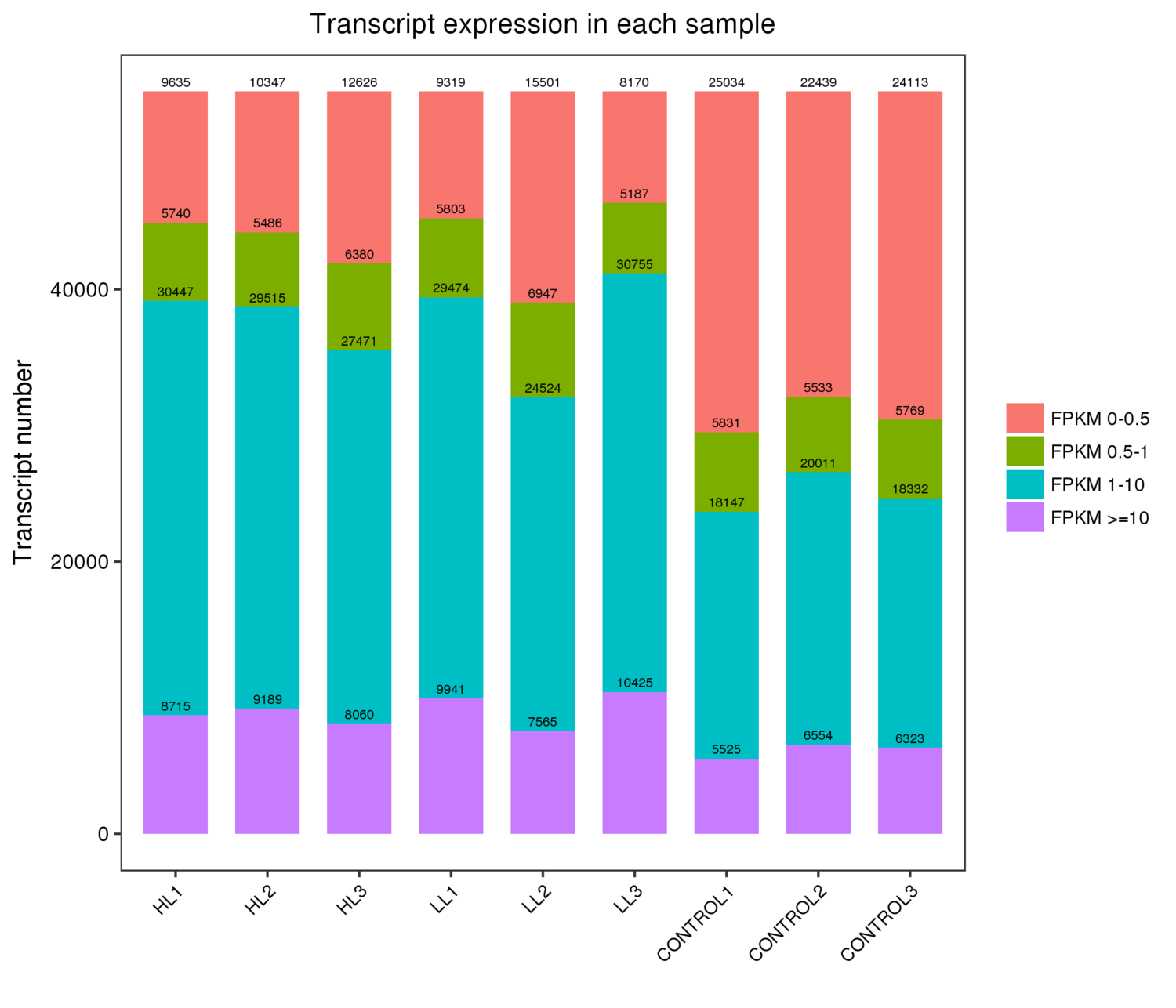


**Fig. S1 Distribution of FPKM value in each sample.** Different colors represent different range of FPKM values. The abscissa is the sample, and the ordinate is the number of Unigene.
